# A decade of fluvial morphodynamics: relocation and restoration of the Inde River (North-Rhine Westphalia, Germany)

**DOI:** 10.1186/s12302-018-0170-0

**Published:** 2018-10-26

**Authors:** Anna-Lisa Maaß, Verena Esser, Roy M. Frings, Frank Lehmkuhl, Holger Schüttrumpf

**Affiliations:** 1 Institute of Hydraulic Engineering and Water Resources Management, RWTH Aachen University, Mies-van-der-Rohe-Straße 17, 52056 Aachen, Germany; 2 Department of Geography, RWTH Aachen University, Templergraben 55, 52056 Aachen, Germany

**Keywords:** River relocation, River restoration, Fluvial morphodynamics, Heavy metal tracer, Fine sediment transport, Morphodynamic equilibrium

## Abstract

**Background:**

Relocations and restorations do not only change the ecological passability and sediment continuity of a river but also its flow behavior and fluvial morphodynamics. Sediment transport processes and morphological development can be assessed with field measurements, also taking the transport of sediment-bounded contaminants as a tracer material for fluvial morphodynamics into account. The objective of this study was to determine the morphological development of the Inde River (a tributary of the Rur River in North-Rhine Westphalia, Germany) towards its pre-defined guiding principle after a relocation and restoration in 2005 AD.

**Methods:**

The fluvial morphodynamics of the Inde River were analyzed over a period of almost 15 years taking sediment samples, analyzing echo soundings of the river’s bathymetry and determining the heavy metal content of the sediment as a tracer material for the morphological development.

**Results:**

The results show that the relocation and restoration of the Inde River initiates new hydrodynamic processes, which cause morphological changes of the river widths, meander belts and channel patterns. The riverbed of the new Inde River has incised into the ground due to massive erosion, which has led to increased fine sediment transport in the downstream direction. The reasons for and consequences of this fine sediment transport are discussed and correlated to the sediment continuity of a river.

**Conclusions:**

Overall, the new Inde River has reached its goal of being a natural river as a consequence of the relocation and restoration and has adapted its new conditions towards a dynamic morphological equilibrium.

## Background

Historically, anthropogenic impacts to river-floodplain systems such as stabilizing and fixing the riverbed, straightening the river course or fixing the riverbanks have heavily modified river-floodplain systems [1–3]. Consequences include changes to the natural morphological development, the sustainable disturbance of the natural bank and bed dynamics, a deterioration of the water quality, changes to the groundwater dynamics of the floodplains, loss of flooding areas and loss of habitats for aquatic and terrestrial ecosystems [1]. Contaminants might be adsorbed on fine-grained sediments [4–6] and thereafter transported downstream with the water phase or deposited on the riverbed or on the floodplains from where they might be remobilized by subsequent flood events [7–9]. River modifications at contaminated sites can have negative effects on the distribution of contaminants or positive effects due to the improvement of the chemical and ecological conditions [10, 11].

In the twentyfirst century, national and international requirements and laws define that these previous anthropogenic impacts should be undone in favor of a natural development of river-floodplain systems. It is stated in the European Union Water Framework Directive (WFD) that water bodies need to be protected against negative modifications and returned to their natural or near-natural states. The objectives of river restorations are a generation of flooding/retention areas, an increase of flow lengths and flood frequencies to re-activate or activate floodplains and to enrich the ecological diversity and the reduction of anthropogenic barriers and the initiation of a natural river development [1]. The natural characteristics of a river-floodplain system are abstracted and formulated in a pre-defined guiding principle, which considers irreversible anthropogenic impacts such as alluvial clay and colluvial sediments, mainly from medieval times, mining-induced activity, urbanization and buildings [12]. In addition, the morphological development, the ecological passability for aquatic organisms and the sediment continuity are two main advantages of restorations [12–14]. Pre-defined development goals should be realized within river restorations and are evaluated by comparing the current state of a river with its guiding principle.

It is always questionable if a restored river reaches its pre-defined guiding principles, how its morphological development takes place and how long it takes to adapt to new conditions. Each river reacts differently to new geometrical, hydraulic and sedimentological conditions, so that it is always reasonable to assess extensively the fluvial morphodynamics of a restored river reach. Thus, extensive field measurements and interdisciplinary cooperation can be helpful to identify, quantify and analyze such morphological adaptation processes.

**Fig. 1:**
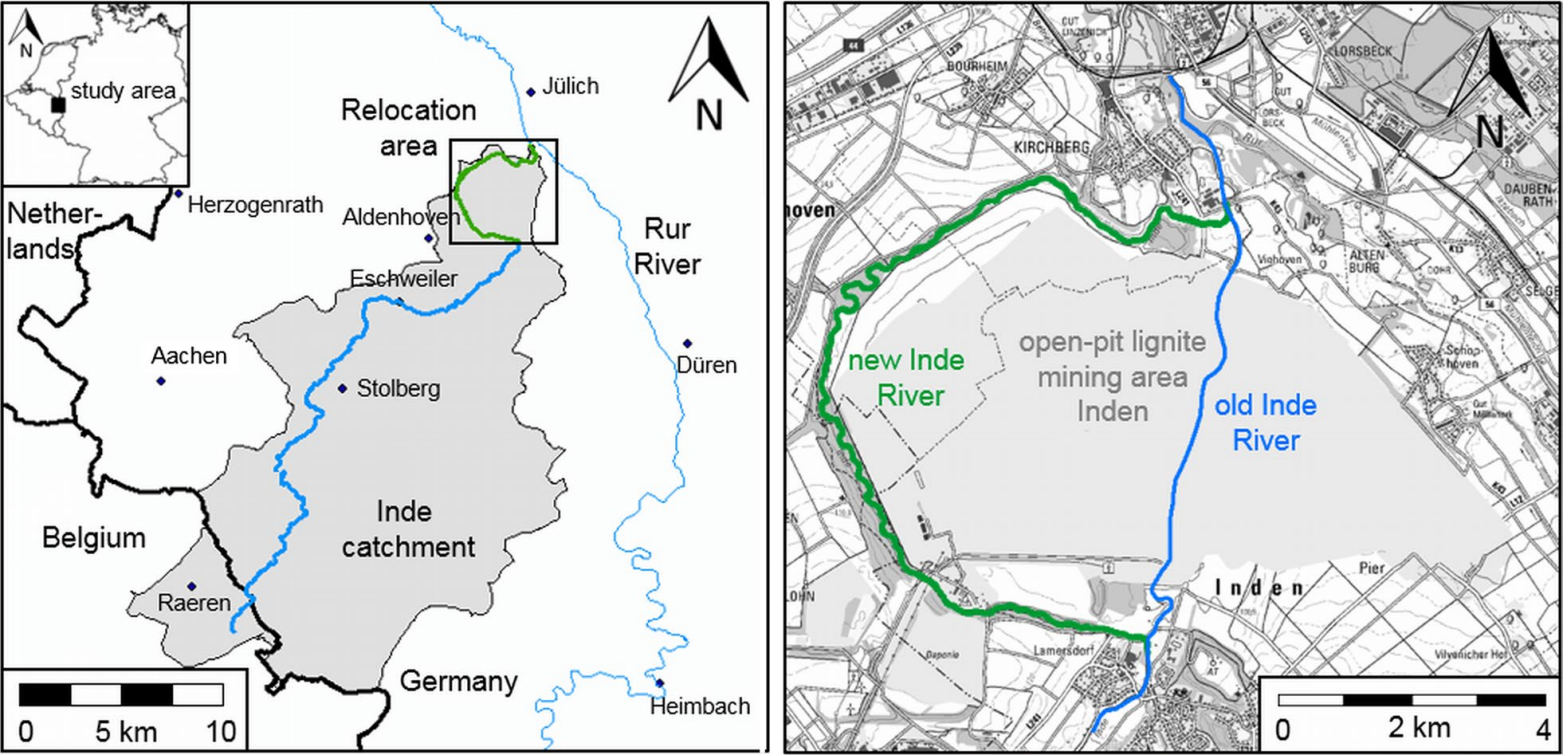
Catchment area of the Inde River (left) and relocation area (right)

The objective of this study was to determine the fluvial morphodynamics of a 13-km-long relocated and restored river reach of the Inde River (a tributary of the Rur River, North-Rhine Westphalia, Germany; see Fig. 1). The fluvial morphodynamics of the new Inde River were determined with extensive field measurements between 2005 AD and 2018 AD. Sediment samples from the riverbed for sedimentological characterization, sediment traps, echo soundings and the adjacent hillsides were analyzed, focusing on the initial morphological development of the new Inde River after its hydraulic connection to the existing river reaches. Additionally, sediment-bounded heavy metals were used as a tracer material to describe the morphological evolution of the relocation and restoration. Heavy metals are persistent substances that can be transported, relocated and accumulated with sediments [15–18]. Anthropogenic activity is one reason for a significant increase in the heavy metal concentrations above the natural geogenic background [19], which is especially present in old industrial regions such as the Inde River catchment [20]. Therefore, heavy metals adsorbed on fine-grained sediments were used as a tracer material to analyze the ongoing morphological development of the Inde River in the new restorations reach.

River restorations or even entire river relocations are complicated and expensive to plan and implement. Due to the fact that morphological changes can always have positive as well as negative consequences, it must be determined if such an intervention will improve the morphodynamics and the river-floodplain system as an environmental habitat. Here, the Inde River is used as a case study to analyze these consequences extensively and to learn from this example for future relocations and restorations. Initially, the results of different analyses of sediment samples and echo soundings of the Inde River are presented. Then, the results of the Inde River, focusing on the temporal scale of its fluvial morphodynamics, and its continuous sediment transport, are discussed and compared to its pre-defined guiding principles.

## Case study area

The Inde River is a 54.1-km-long river in North-Rhine Westphalia in Western Germany with a catchment area of 344 km^2^. After a flow length of 2.5 km from its source in Belgium, the Inde River reaches the Belgian–German border and flows south of the city of Kirchberg into the Rur River. The main tributaries are the Vichtbach and the Wehebach. Parts of the Inde River and its tributaries have been straightened and canalized [21–23]. Two reservoirs are in the Inde catchment. The Wehebachtalsperre reservoir was constructed at the Wehebach (1977–1981) and the Dreilägerbachtalsperre at the Dreilägerbach (tributary of the Vichtbach) (1909–1912) to ensure a drinking water supply [22–24]. Open-pit lignite mining is present at the Inde River. In the mining areas, the continuous pumping of groundwater is necessary to dry out the open-pit lignite mining areas to enable uninterrupted mining. Thus, mine water from the open-pit lignite mines is partly passed into the Inde River close to the communities of Lamersdorf (on average 1.0 m^3^/s) and Kirchberg (on average 0.5 m^3^/s) and increases the average low water discharge of the Inde River [25]. The average low water discharge of the Inde River is 0.54 m^3^/s. The mean annual discharge is 2.82 m^3^/s and the highest annual discharge is 89.48 m^3^/s (1965–2016, gauging station Eschweiler) [26]. Generally, the widespread pumping of ground water leads to decreased flood discharges, while the high gradients and heavy rainfalls in the upper part of the Inde River result in sudden flood events especially during the wintertime [27].

Devonian bedrock is predominant in the upper reaches of the Inde River. The middle reaches are characterized by carbonates of the Carboniferous. In the lower reaches, the river flows through the Lower Rhine Embayment with Tertiary and Quaternary sediments [27, 28]. In the upper part, the Inde River is characterized as a small coarse substrate dominated siliceous highland river (German Stream Type 5). Between Walheim and Stolberg, it is characterized as a small coarse substrate dominated calcareous highland river (German Stream Type 7). Between Stolberg and Eschweiler, the Inde River is defined as a mid-sized fine to coarse substrate dominated siliceous highland river (German stream type 9), whereas downstream of the city of Eschweiler until the mouth of the Inde River, it is characterized as a mid-sized and large gravel-dominated lowland river (German Stream Type 17) [29].

**Fig. 2:**
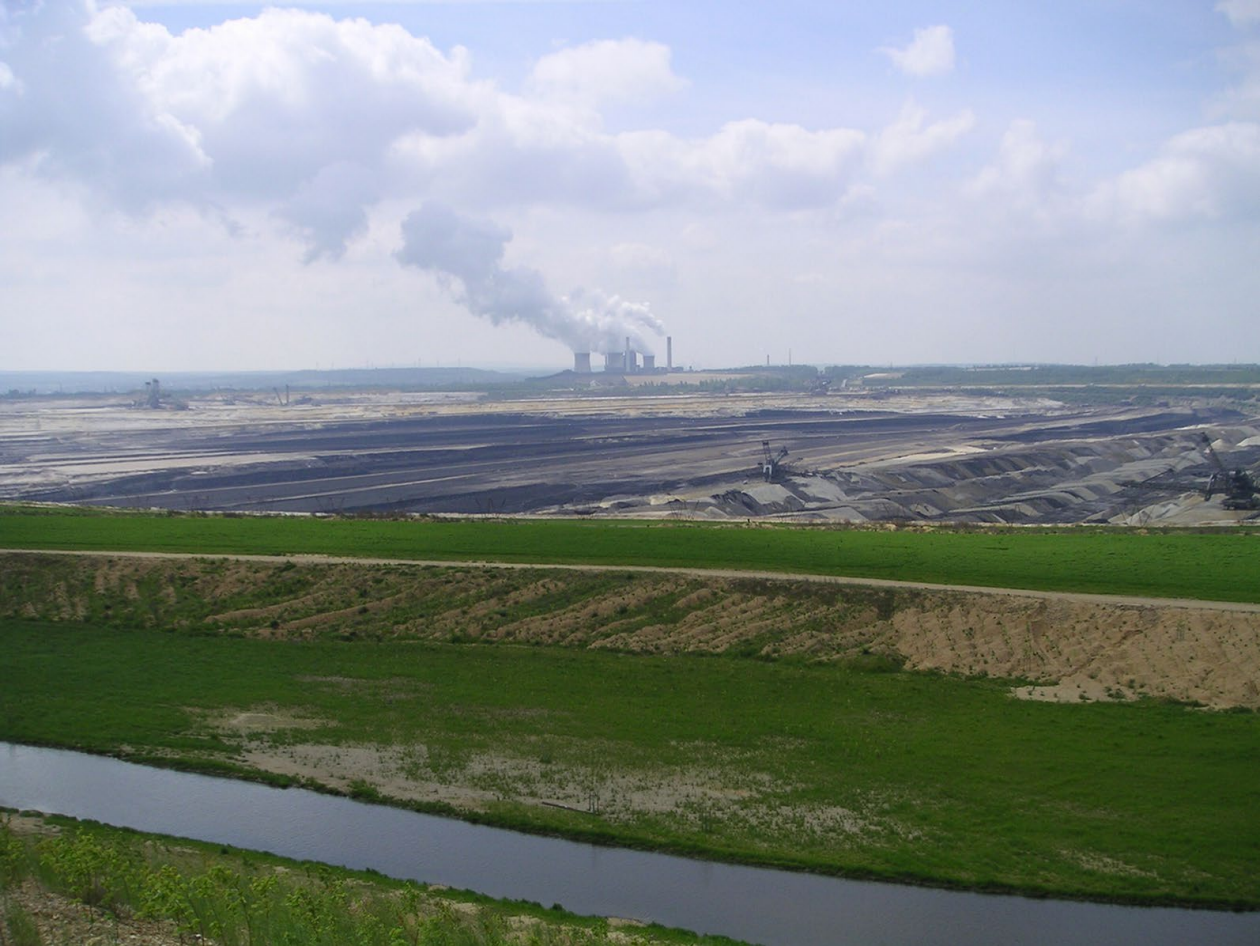
Open-pit mine Inden with the Inde River (foreground) (Photo: IWW, RWTH Aachen)

Until 2005 AD, the lower reaches of Inde River flowed through the prospective extraction field of the open-pit lignite mining area Inden, which belongs to the Rhenish lignite-mining region in Western Germany between the cities of Aachen and Mönchengladbach (see Fig. 2). A -km-long reach of the Inde River (old Inde River) was relocated as the consequence of the progressing open-pit lignite mining towards a 12-km-long river reach (new Inde River). The relocation includes different restoration interventions. The project was finished in 2005 AD, and the system was hydraulically connected to the existing river reaches upstream and downstream (see Fig. 1).

The design of the new part of the Inde River followed a guiding principle that was pre-defined based on the natural characteristics of the Inde River [30], considering some restrictions of the adjacent and ongoing open-pit mining. Realizing the guiding principle means an improvement of the current river state taking spatial, geological and other restrictions into account. Here, the restrictions are the stability of the slope of the open-pit mine, the flood protection as well as the ongoing progress of the open-pit lignite mining. It defines the geographical position of the river relocation in its spatial and temporal limits. The additional discharge of mine water into the Inde River (in Lamersdorf, approx. 1.0 m^3^/s, to an average low-water discharge of 0.54 m^3^/s) as well as the location of a noise abatement dam and the requirement that the riverbed should consist of natural ground surface material from the open-pit mine, restricts the restoration. The natural ground surface material (so-called Forstkies) consists of a mixture of loess and gravel (silty loam) with a *D*_15_ of 0.06 mm, a *D*_50_ of 0.4 mm and a *D*_90_ of 3 mm [30]. Limitations of the artificial construction, using large spreaders for coal mining, without leveling to prevent soil compaction, lead to a rough and undulate surface [31]. Vertical erosion is restricted to an artificially built sealing layer, which consists of a mixture of mineral soil materials [32]. The sealing layer restricts the infiltration of water into the ground to a certain limit, which would otherwise counteract the continuous pumping of the groundwater mentioned above. Additionally, lateral erosion is only possible in a defined development corridor with a width between 70 and 400 m [32]. Further limitations are the adjacent forests south of Kirchberg and the smooth transitions of the river width, depth and slope at the upstream and downstream ends of the new river reach to the old river course.

In former times, the presence different geo-resources in the catchment area of the Inde River resulted in the widespread mining and treatment of iron, lead and zinc ores close to the city of Stolberg. In addition, underground coal mining and open-pit lignite mining occurred especially close to the city of Eschweiler [8]. These anthropogenic activities led to increase in the natural, geogenic background concentrations of heavy metals, which are distributed in the landscape over different input paths [33]. Although neither mining nor most of the metal industry exist in this area anymore [34], high heavy metal concentrations from historical times can still be found in this region [27]. The water quality of the Inde River is affected by these increased concentrations [23].

## Methods

The field measurement campaign started in 2005 AD, after the completion of the relocation and restoration of the Inde River, and ended in 2018 AD. The campaign is subdivided into two parts: (1) For the years 2005 AD until 2012 AD, sediment samples from the riverbed were collected for sedimentological characterization, and sediment traps, echo soundings and the adjacent hillsides were analyzed. The focus was on the initial morphological development of the new Inde River after its hydraulic connection to the already existing river reaches. (2) Different sediment samples from the riverbed of the years 2017 AD and 2018 AD as well as suspended sediments sampled during a bankfull discharge were analyzed, focusing on its heavy metal content as a tracer for the ongoing morphological development of the Inde River in the restoration reach.

**Fig. 3:**
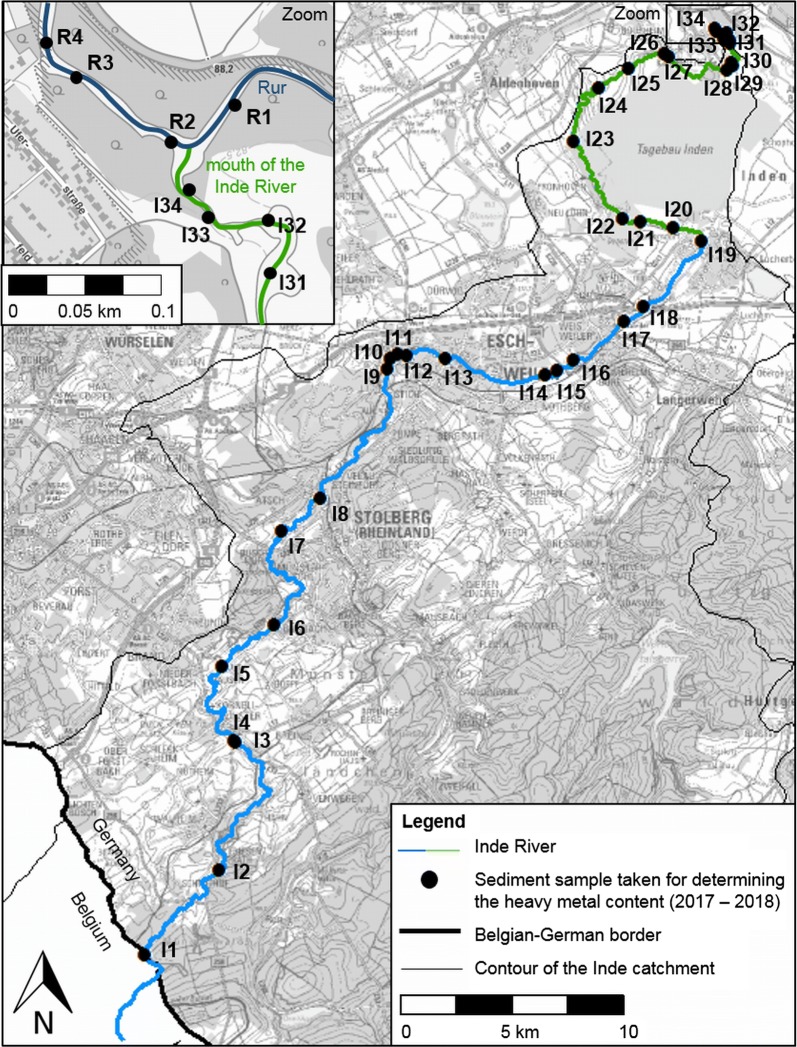
Sediment samples taken from the whole Inde River for determining the heavy metal content

**Fig. 4:**
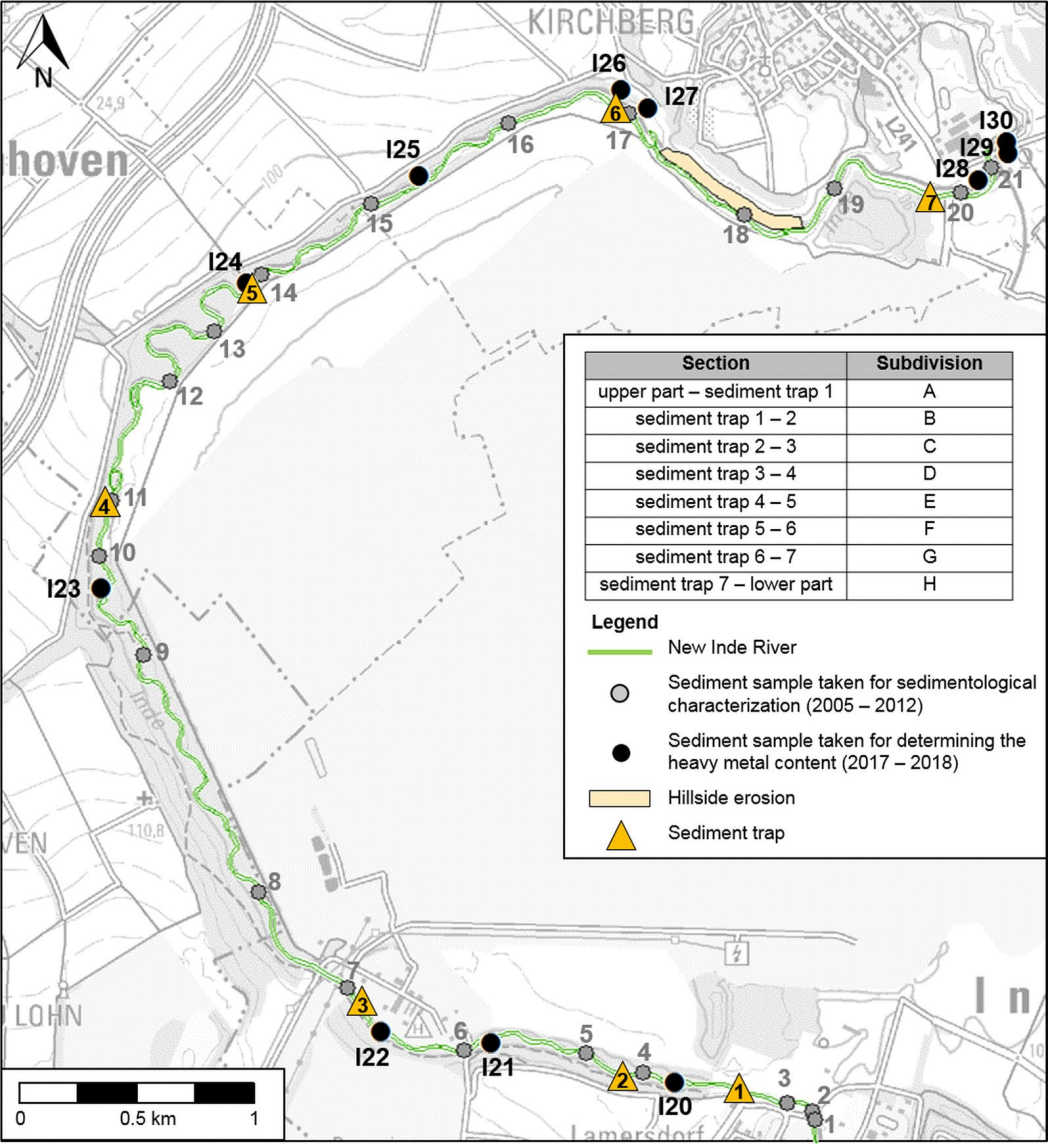
Field measurement locations inside the new Inde River reach between 2005 AD and 2018 AD

The first field measurement campaign (1) ended in 2012 AD because the results of the analyses showed that the new Inde River had developed towards its guiding principles and had adapted the new hydrodynamic and morphodynamic conditions. Significant changes were no longer observed. The morphological development after an additional period of 5 years was investigated in 2017 AD and 2018 AD to obtain a temporal large-scale overview of the fluvial morphodynamics of the new Inde River over an entire period of almost 15 years. Such a large-scale consideration also enables the analysis of the morphological connection of the restored river reach to the upstream and downstream older river reaches. Divided according to the different campaigns, Figs. 3 and 4 show all field measurement locations during the entire investigation timespan of 2005 AD to 2018 AD.

### Sedimentological characterization

The sediment characteristics of the riverbed were mapped over the entire area. 23 sediment samples were taken from the riverbed: 18 samples from the new river reach, two samples from the old river reach upstream of the restoration, two samples from the Rur River and one sample from the hillside adjacent to the new Inde River at the left bank almost at the end of the new course (see Fig. 4). Because of the sedimentological variety of the new Inde River, the sediment samples were not equally distributed over the entire new river reach. Four sediment samples were taken from the sand-dominated parts (grain size diameter between 0.063 and 2 mm), eight samples were taken from the gravel-dominated parts (grain size diameter between 2 and 63 mm) and six samples were taken from the gravel-dominated parts with a proportion of more than 5% stones (grain size diameter larger than 63 mm). All sediment samples were dried at 105 °C and sieved to determine their grain size distribution.

The bank dynamics were mapped considering the steep erosion of the riverbanks or banks inside the watercourse. The hydrometry was also mapped, focusing on rapids, tributaries and branches.

### Sediment traps

During the construction phase, seven structurally identical sediment traps were installed in the new Inde River reach (see Fig. 4). Sediment traps are basin-shaped extensions or depressions located in the river, which are permanently passed through by water and sediment [35]. Such a sediment trap traps incoming bed load and balances, in the case of the new Inde River reach, the input and output of the bed load towards an equilibrium. The hydraulic function of the sediment traps is regulated by a reduction of the current shear stress caused by reduced flow velocities. The reduced current shear stress consequently results in the deposition of sediment inside the sediment trap.

At the beginning of the measurements between 2005 AD and 2006 AD, the continuous transport of the bed load in the downstream direction was limited because of these sediment traps. In 2006 AD, an event with a discharge of 26 m^3^/s and flow velocities of approximately 0.5 m/s occurred in the Inde catchment. Such an event occurs statistically five times in 1 year, especially during summer periods [36]. The sediment volume trapped in the seven sediment traps before (reference date August 2005 AD) and after the flood event (March 2006 AD) was compared, and the sedimentological composition of each trap was analyzed. The amounts of sediment deposited in the sediment traps were also analyzed and conclusions regarding the stability of the different river reaches between two sediment traps were drawn. Trapped material can be returned into the system and potentially compensate for the vertical erosion of the riverbed [37]. Today, all sediment traps are emptied irregularly, which allows the continuous transport of sediments inside the restoration area.

### Analysis of echo soundings

**Fig. 5:**
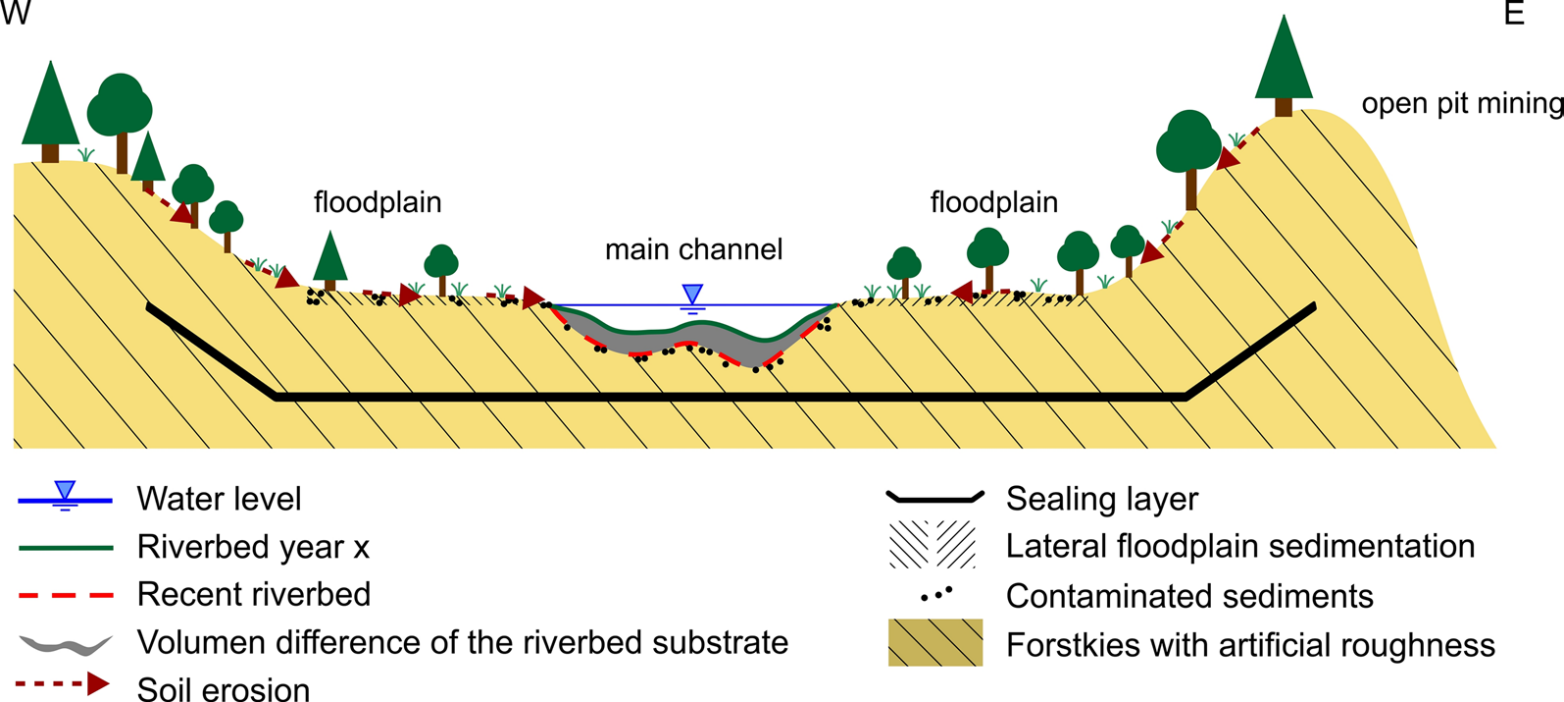
Illustration of erosion and accumulation processes in the riverbed and on the floodplains

The erosion and sedimentation rates were determined from echo soundings. The riverbed elevation of the new Inde River was determined every year between 2005 AD and 2012 AD based on echo soundings. In several cross-sections of the new Inde River, echo soundings were performed. Based on the deepest point of the sounding data of each cross-sectional profile, the longitudinal profile of the new Inde River was determined. The echo soundings were used to quantify the temporal morphological development of the restoration reach of the Inde River between 2005 AD and 2012 AD. The erosion and sedimentation rates of each cross-sectional profile as a function of time were determined for different subdivided sections (see sections in Fig. 4). To determine the volume of erosion or sedimentation for one section, the sounding data of one cross-sectional profile are seen as a representative data set for the adjacent river section. The erosion and sedimentation rates were quantified by comparing the elevation of the riverbed in a cross-sectional profile of 1 year to the elevation of the same profile of another year (see Fig. 5). When sounding data were missing, which is the case between 2010 AD and 2012 AD, the erosion and sedimentation rates could not be determined for all subdivisions of the new Inde River. The maximum erosion depth of the riverbed was determined by analyzing the distance between the riverbed elevation measured with echo soundings and the sealing layer, which was set as an erosion base during the construction phase. The echo soundings were also used to determine the local erosion of the riverbed and to quantify the potentially hazardous distances to the sealing layer. The maximum depth of erosion was equal to the deepest point of each cross-sectional profile. This depth was compared to the elevation of the sealing layer to determine the minimum vertical distance between the riverbed and the sealing layer.

### Hillside erosion

**Fig. 6:**
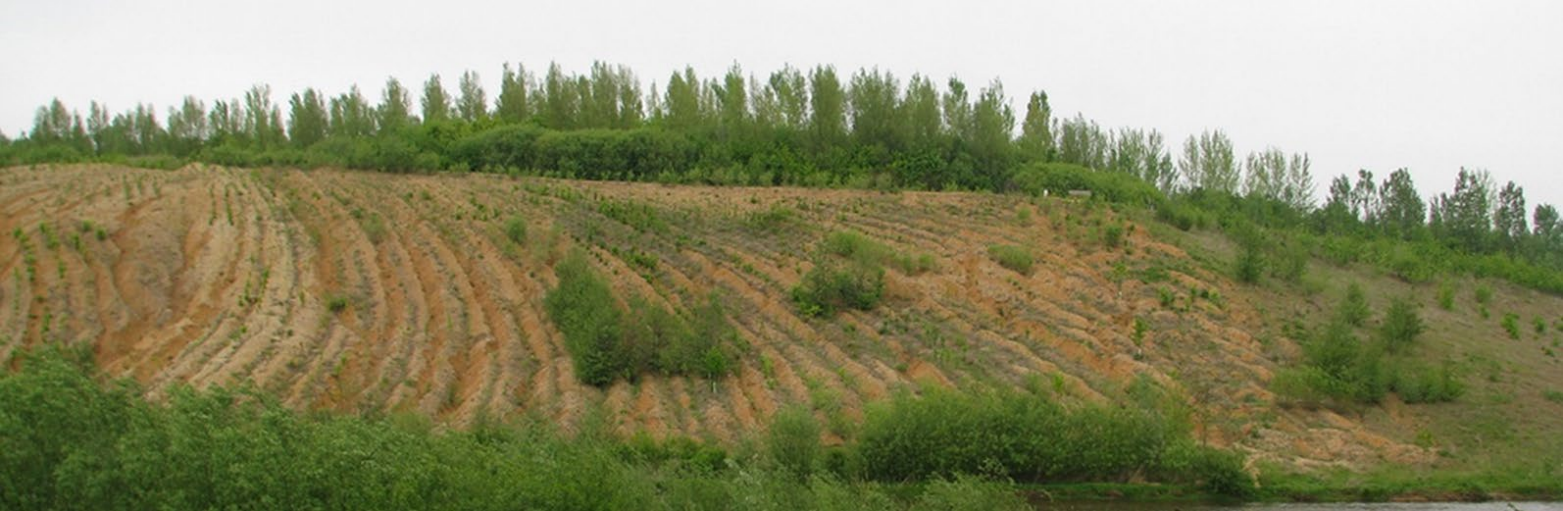
Wave structure with gullies of the hillside (Photo: IWW, RWTH Aachen)

One hillside, adjacent to the new Inde River at the left bank, almost at the end of the new course (see Fig. 4), showed significant soil erosion contributing to sediment transport. During the construction phase, the ground surface of the hillside was formed using a spreader for coal mining by a wave structure with gullies (see Fig. 6). Rainfall events led to significant hillside erosion through these gullies into the Inde River (see Fig. 5).

**Fig. 7:**
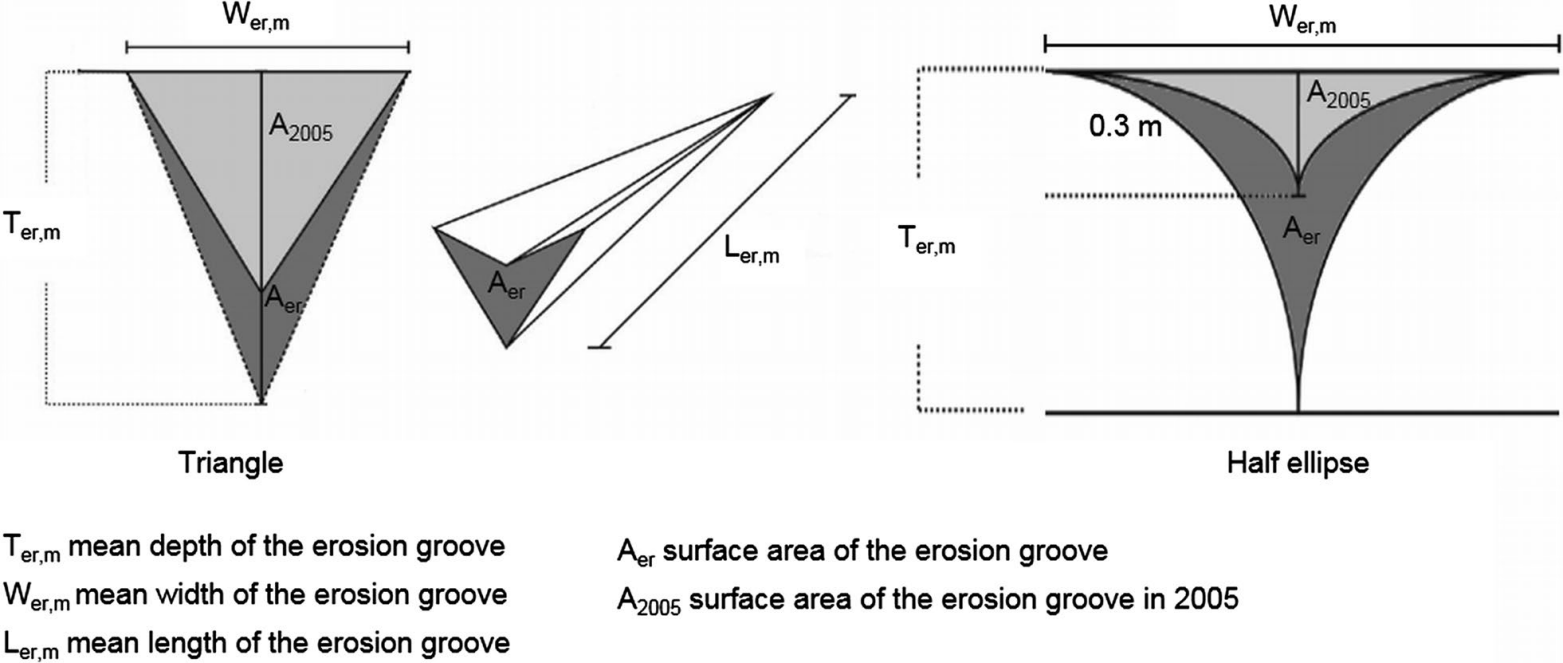
Simplification of the structure of the erosion gully (modified after [41])

The amount of hillside erosion was determined by simplifying the gully surface area to two different geometrical structures: (1) a triangle and (2) a half ellipsis (see Fig. 7). The number of erosion gullies was further counted and their depths and lengths were measured, and it was determined if the gullies had a connection to the riverbed or not. Under the assumption that the depth of the gullies is linear to the depth at the upstream end, which is the depth after finishing the restoration, the body of the eroded material in each gully is prismatic.

### Using heavy metals as a tracer for determining fluvial morphodynamics

The content of heavy metals adsorbed on the sediment particles of the samples were further analyzed and used as a tracer to determine the morphodynamic development of the new Inde River. Heavy metals can enter into the fluvial system by different processes such as weathering, erosion and transport as well as anthropogenic activities. There, hydrological processes are the driving force for their dispersion [38]. Heavy metals occur in the dissolved and particulate phases depending on different factors such as chemical parameters. Nevertheless, they are mainly bound on fine-grained sediments, especially silt and clay (< 63 µm). Thus, sediments represent an important natural transport medium for heavy metals in fluvial systems, which is controlled by recent and past morphodynamic processes such as erosion, transport and accumulation [39]. Due to the fact that the restoration of the Inde River consists of a 13-km-long new riverbed, which was constructed with substrate [Forstkies, mixture of gravel and loess (silty loam)], showing lower geogenic background concentrations, it is possible to use sediment-bound heavy metals as a tracer for determining and analyzing the natural sediment transport and the ongoing morphological development of the Inde River in the new restorations reach.

In 2017 AD and 2018 AD, samples of the surface layer, which represents the morphologically active substrate of the riverbed, and composite samples of the upper 20 cm of the riverbed, were taken from the whole Inde River (see Fig. 3, samples I1–I34). Additional samples were taken in the Rur River close to the mouth of the Inde River (see Fig. 3, samples R1–R4). The composite samples of the upper 20 cm were sampled using a riverside auger (stainless steel) with a diameter of 6.5 cm. The samples of the surface layer were sampled using a cone-shaped pot (height: 24 cm, diameter of opening: 14 cm, stainless steel).

To determine the impact of flood events on the heavy metal distribution in the new riverbed, high discharges were artificially simulated within the river channel (method after [40]). A cylinder with a height of 90 cm and a diameter of 55 cm was placed on the riverbed to create an enclosed water column inside the river. Then, by whirling up the water and the surface of the riverbed, the sediment was remobilized. The suspended sediment was sampled as a water sample of 20 l at each sampling site. After a settling time of 1 week, the sediment was separated from the water. These sediment samples represent the fraction that can potentially be remobilized from the riverbed during high discharges.

In addition, one water sample with suspended sediment was taken during a bankfull discharge of approximately 15 m^3^/s upstream of the new river reach (same sampling site as I19, see Fig. 3). This sample was treated like the samples taken with the method after [40].

Furthermore, four samples of the natural surface material (Forstkies) were taken in the surroundings of the river to compare its natural background of heavy metal with the content of the riverbed sediments of the new Inde River.

To determine the heavy metal content, all sediment samples (fractions < 63 µm) were dried at 105 °C and analyzed with X-ray fluorescence (XRF) analysis.

## Results

### Channel pattern and sedimentology

**Fig. 8:**
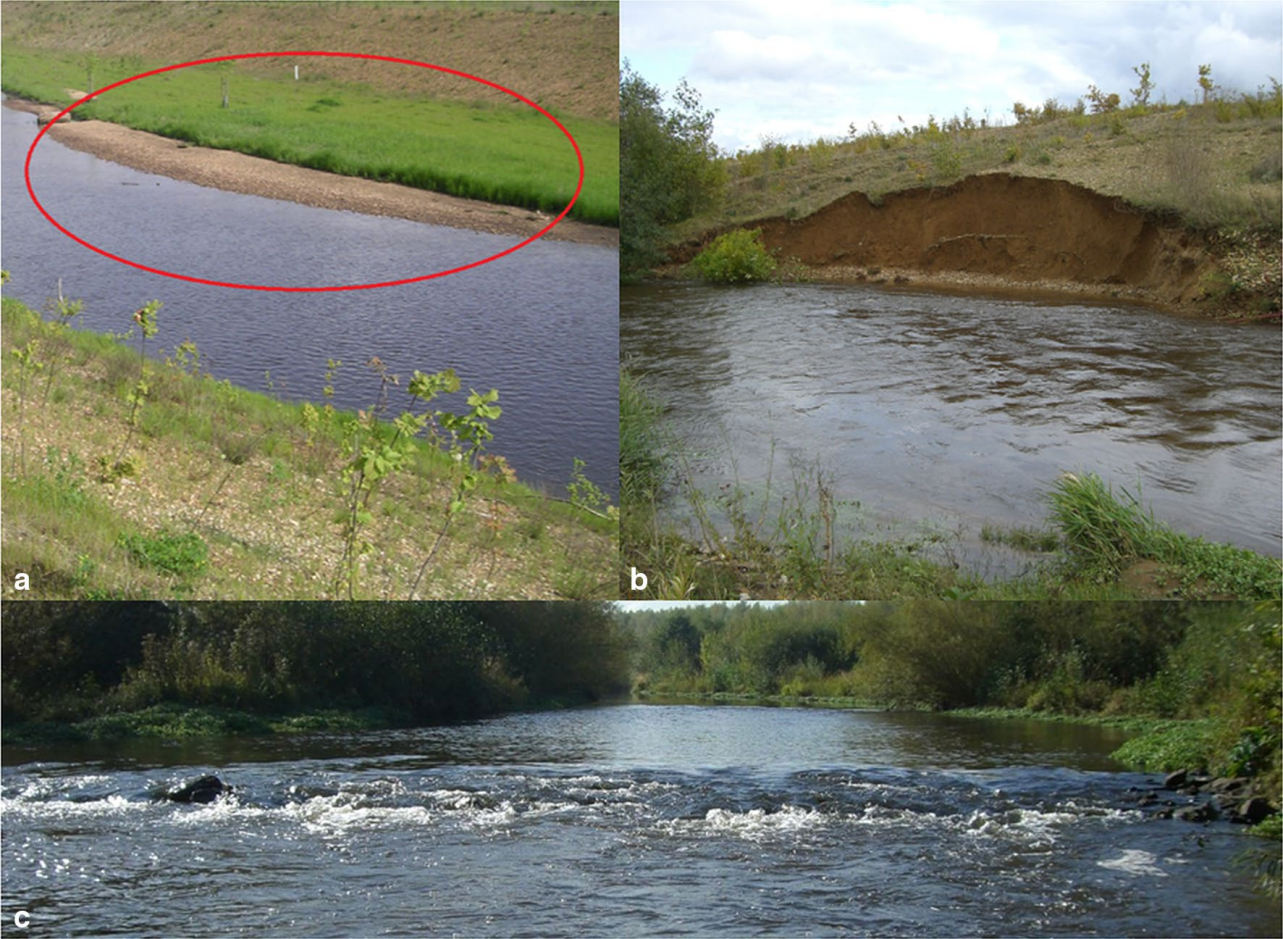
**a** Sediment bank, **b** steep riverbank and *c* rapid of the new Inde River [41]

Sediment banks develop in areas with low flow conditions [41] (see Fig. 8). The creation of small groynes leads to changes of the main flow direction, which result in cut banks and slip-off banks and a high meander migration potential.

**Fig. 9:**
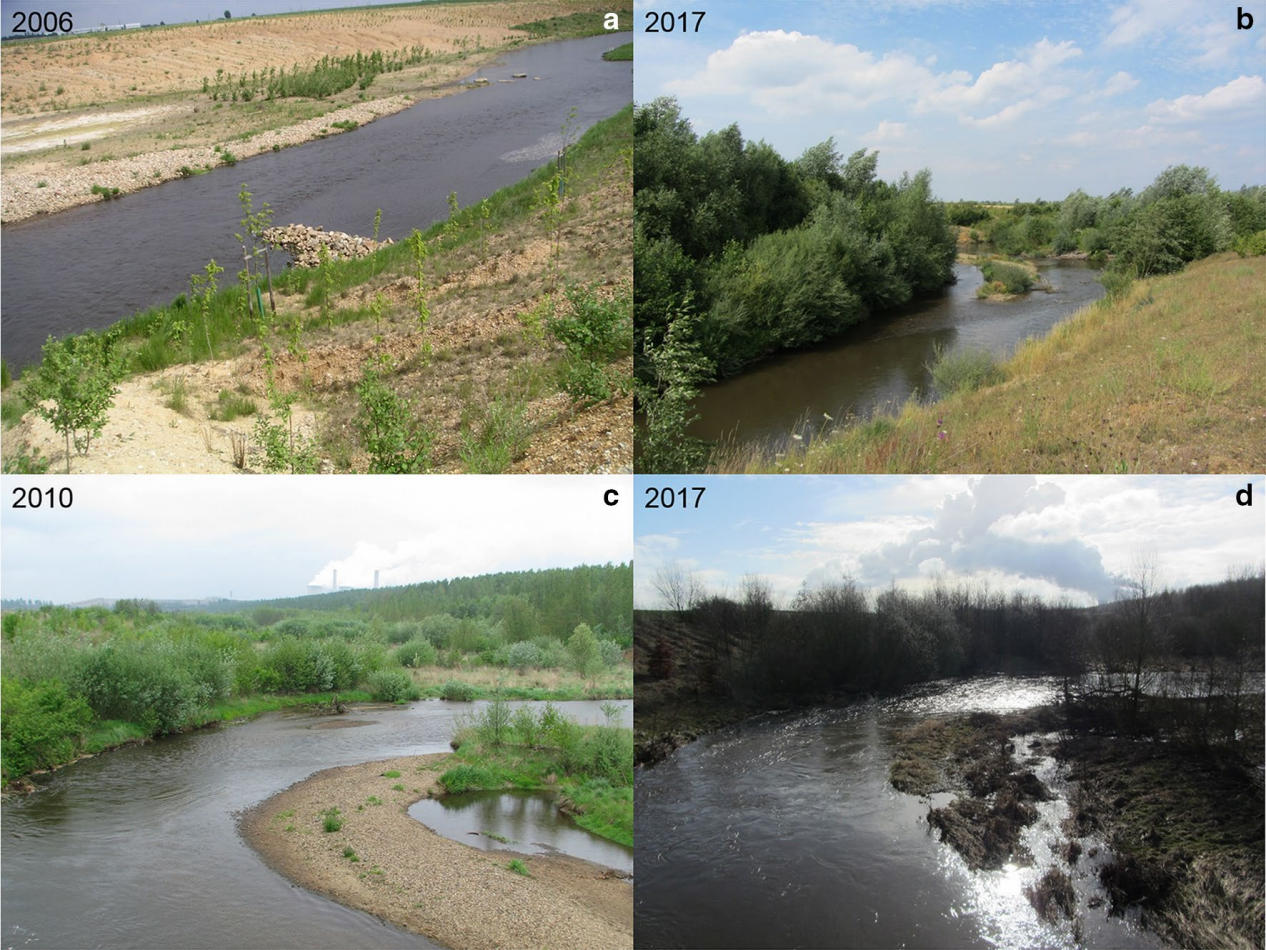
**a**, **b** Comparable river sections. **c**, **d** Same river section during low flow conditions and during a flood event (**a**, **c** photos IWW, RWTH Aachen; **b**, **d** photos PGG, RWTH Aachen)

In 2006 AD, a clear artificial structure of the adjacent hillsides, banks and groynes can be seen. In 2017 AD, the hillsides and the banks and groynes are more overgrown. An accommodated flow diversity is present (see Fig. 9).

In addition to the geometrical adaptation, a sedimentological adaptation takes place. After the completion of the restoration in 2005 AD, the riverbed consisted predominantly of sand and clay, which erodes at small shear stresses and is transported in the downstream direction into the Rur River. The erosion leads to an incision of the riverbed of the new Inde River into the ground, to the formation of an armor layer and to a coarsening and washing out of the fine sediment of the riverbed.

**Fig. 10:**
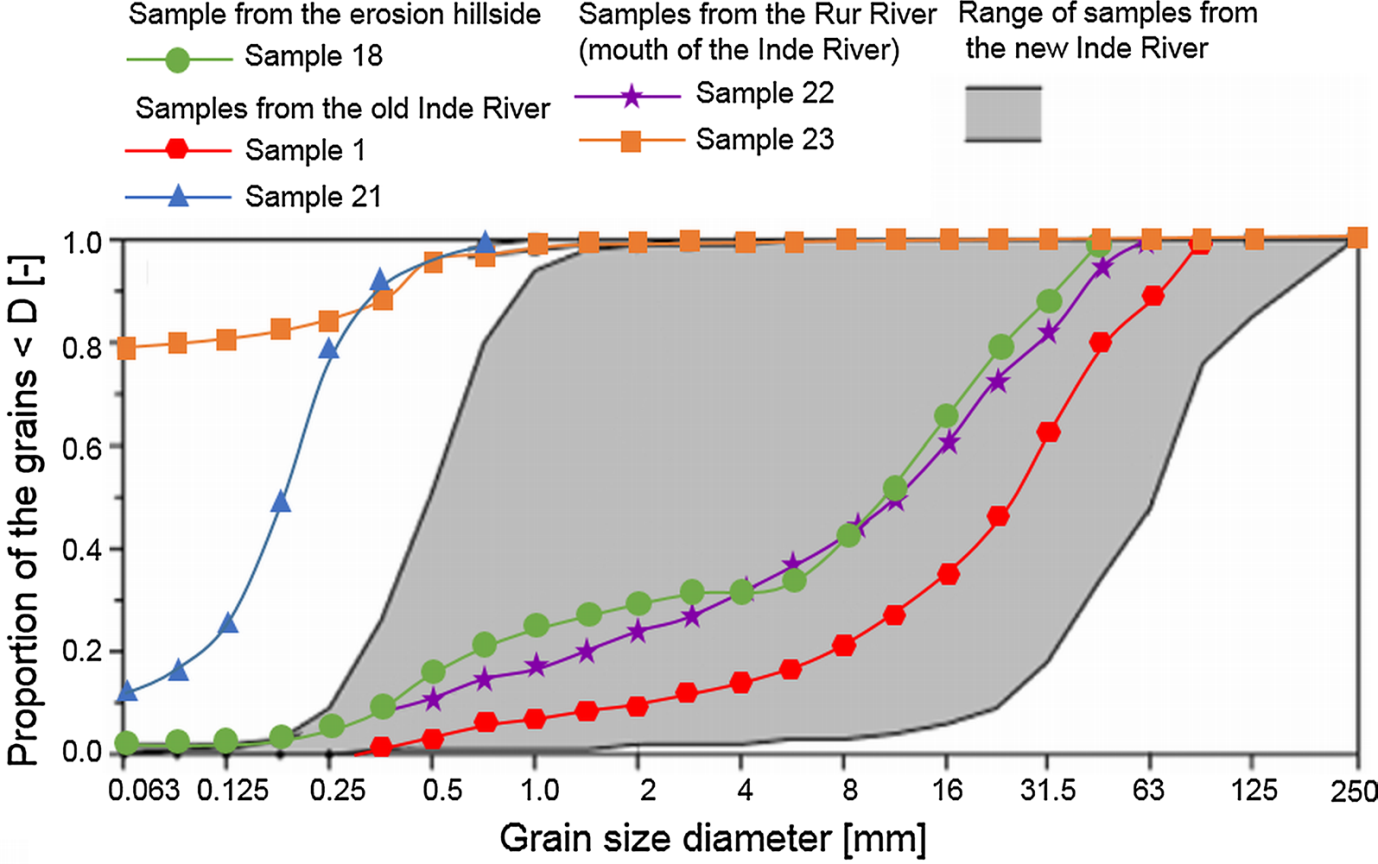
Comparison of the grain size distributions (sample locations refer to Fig. 4) (modified after [41])

The flow velocities and grain size distributions of the riverbed of the new and the old Inde River are variable. A comparison of the grain size distribution of the new Inde River with the grain size distribution of the substrate used for preparing the riverbed (Forstkies) of the new reach in 2005 AD shows that the current riverbed is coarser than in 2005 AD. The riverbed of the new Inde River mostly consists of gravel with a proportion of sand of 20% up to 60%. A sandy riverbed can especially be found in areas with reduced flow velocities behind roughness elements (stones), groynes and meander belts or at slip-off banks. Scour holes with depths up to 1.5 m occur in clayey areas at steep riverbanks. In addition, the grain size distribution of the hillside erosion is coarser than that of the initial substrate and the sediments upstream of the restoration reach are significantly coarser than those downstream of this new reach (see Fig. 10). The riverbed of the Rur River close to the mouth of the Inde River is gravel-dominated like the riverbed of the Inde River itself, but with a higher proportion of fine material [41] (see Fig. 10).

### Erosion and sedimentation rates

**Fig. 11:**
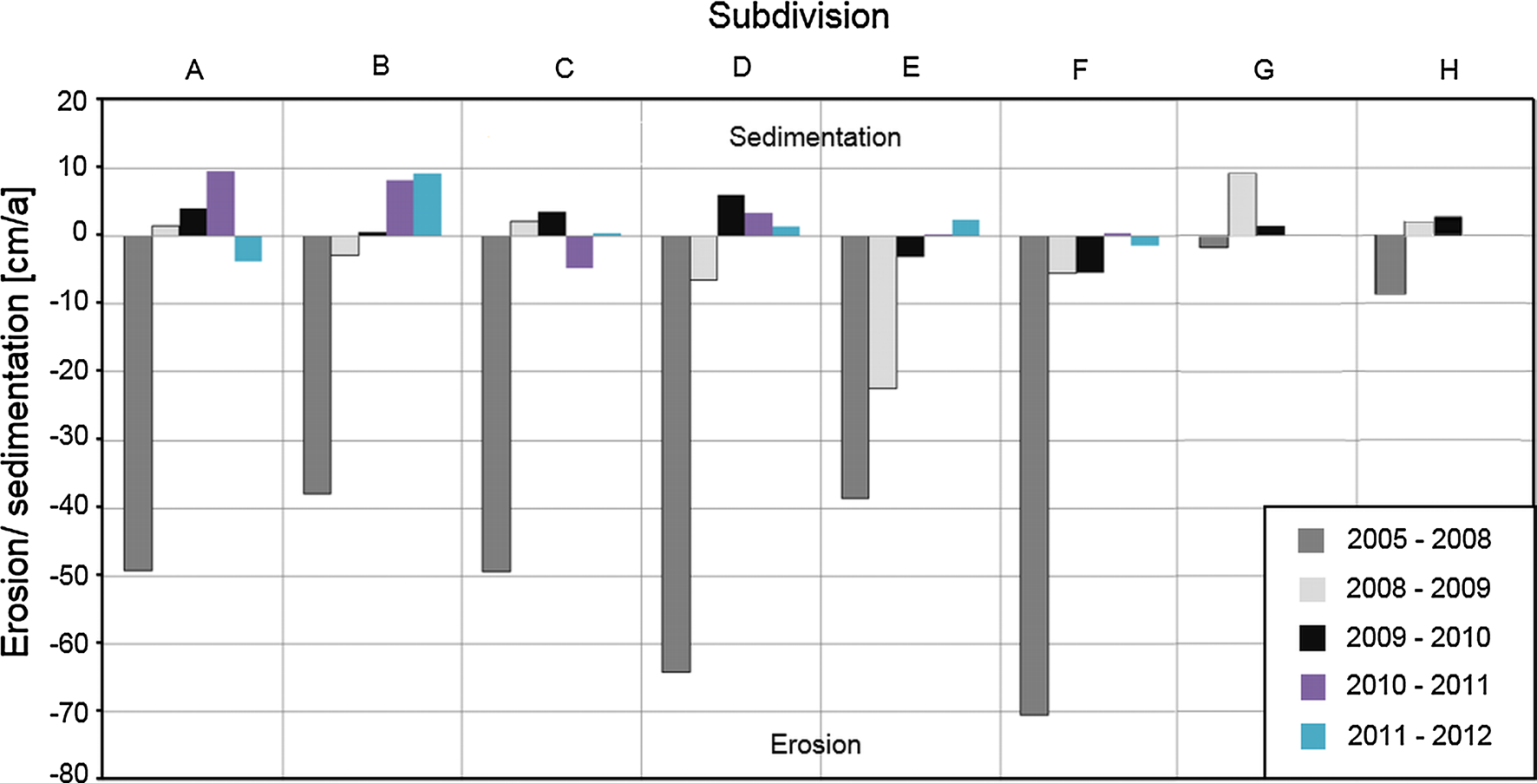
Erosion and sedimentation determined from echo soundings (modified after [41])

Figure 11 summarizes the amounts of erosion and sedimentation for all subdivisions (A until H, see Fig. 4) of the new Inde River reach. There are no data for subdivisions G and H between 2010 AD and 2012 AD.

Significant erosion took place between 2005 AD and 2008 AD. Many flood events occurred in 2007 AD, followed by an extreme flood event in 2008 AD with a discharge of more than 80 m^3^/s. A flood event with a 100-year flood return interval occurs at discharges of approximately 111 m^3^/s [36], so that this flood event has a statistically smaller occurrence interval. This sequence of flood events resulted in the erosion of the riverbed and a high morphological development. Between 2008 AD and 2009 AD, the erosion processes significantly decreased and changed into sedimentation of approx. 400 m^3^. The analysis of the echo soundings of 2009 AD and 2010 AD shows a low erosion of approx. 300 m^3^, which even changes to sedimentation, if the dredging of sediment traps 6 and 7 is considered. Although there are only a few sounding data sets for the years 2010 AD, 2011 AD and 2012 AD, the results show, as expected, a significant decrease of the fluvial morphodynamics of the Inde River compared to those in the first 5 years after finishing the restoration. Only little erosion or even sedimentation is recorded, and the riverbed is on a constant level with a small trend towards sedimentation with an average rate of 2 cm/a [41].

**Fig. 12:**
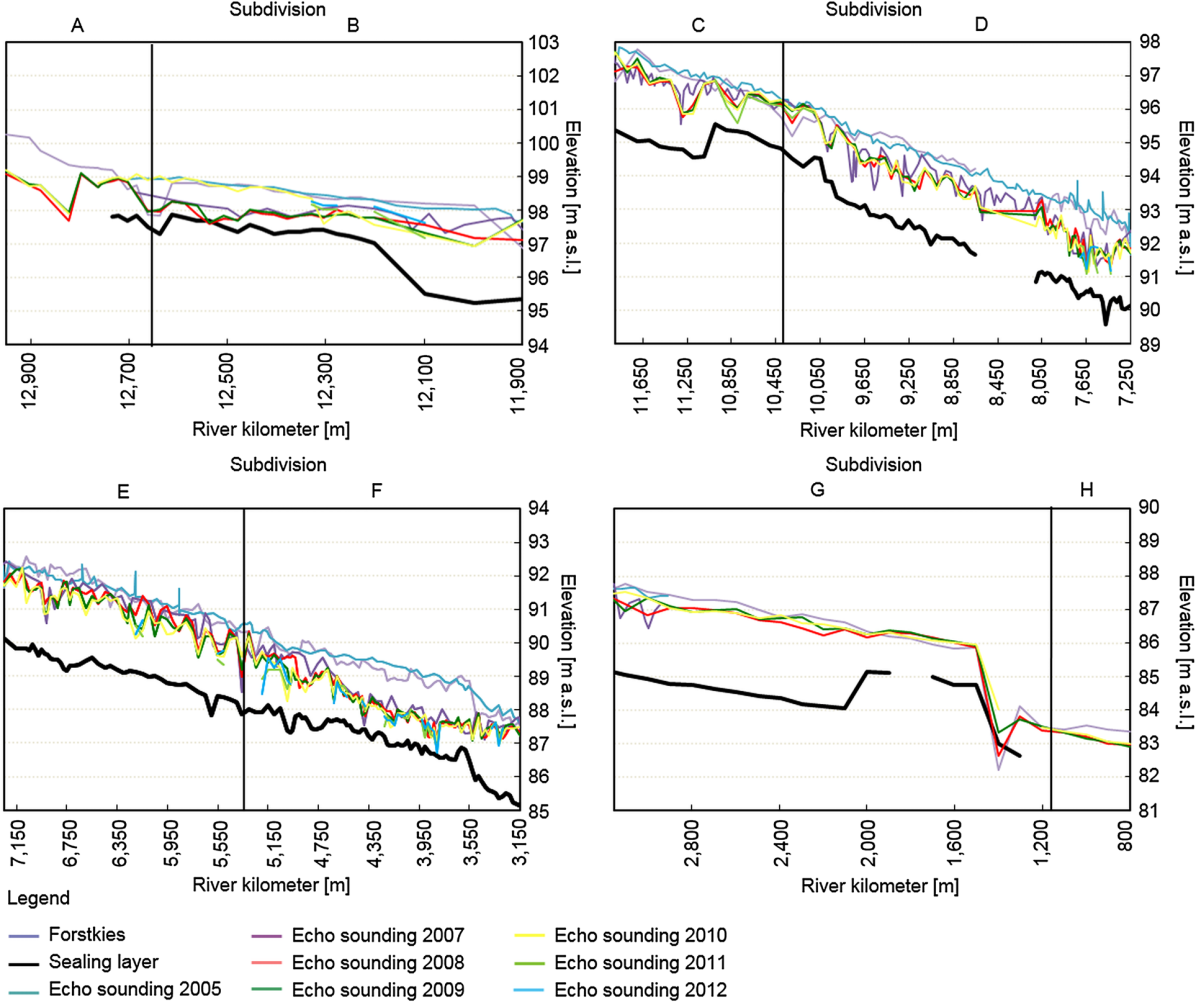
Development of the longitudinal profile of the new Inde River (modified after [41])

Figure 12 shows the longitudinal profiles of the new Inde River between 2005 AD and 2012 AD based on the deepest point of the echo sounding results of each cross-sectional profile. A comparison of the different riverbed elevations reflects the erosion or sedimentation of the riverbed in the longitudinal directions. The massive erosion of sediment in the downstream direction results in the incision of the riverbed into the ground and a significant sediment input into the Rur River. Despite this incision, the riverbed is still located above the sealing layer, which was installed as a local erosion base during the restoration. The vertical difference in height between two echo sounding data sets is at some locations only a few decimeters (state 2012, see Fig. 12). Only at river km 12.5 did the incision reach the sealing layer in 2008 AD and 2009 AD. Therefore, the sealing layer was repaired locally (see Fig. 12).

### Hillside erosion

Under the assumption of a triangular volume of the gullies, an erosion volume of approx. 3500 m^3^ coming from the hillside between 2005 AD and 2012 AD is determined. Under the assumption of a half-ellipse shape, the total erosion volume was approx. 1600 m^3^. Here, it is assumed that the erosion volume was approx. 3000 m^3^ with a deviation of ± 1000 m^3^ [36]. The material eroded from the hillside is characterized with a *D*_10_ of 0.375 mm, a *D*_50_ of 12 mm and a *D*_90_ of 31.5 mm (see Fig. 10). Data on soil compaction, density and porosity were not collected.

The development of the vegetation cover was less than expected for the first 7 years after the completion of the restoration in 2005 AD. The risk of hillside erosion is greater due to the reduced vegetation on the surface. Vegetation would increase the resistance of the soil to erode [36]. Additionally, the gullies increase the roughness of the hillside and result in decreased flow velocities and higher water depths, which also lead to higher shear stresses [36].

### Sediment trapping

Table 1 shows the difference in the sediment trapped in the sediment traps after the event in 2006 AD to the reference date in 2005 AD.

**Table 1:** Amount of sediment and changes respective to grain size fractions in the sediment traps. Modified after [37]

Sediment trap	Amount of sediment (m^3^)	Changes of the gravel fraction (%)	Changes of the sand fraction (%)	Changes of the silt fraction (%)	Changes of the clay fraction (%)
1	70	− 0.1	+ 36	+ 34	− 2
2	315	− 17	+ 9	+ 8	− 1
3	340	− 8	+ 25	+ 10	+ 7
4	475	− 10	− 18	+ 26	− 2
5	80	− 7	+ 25	− 16	− 2
6	860	− 4	− 8	+ 17	− 5
7	70	+ 0	+ 27	+ 65	+ 7

The bed load coming from upstream of the restoration is deposited in sediment trap 1 and reflects the initial sedimentological situation of the Inde River. During the event of 2006 AD, 70 m^3^ of sediment was trapped in sediment trap 1. In sediment trap 2, the amount of sediment was 315 m^3^. Such an increase between sediment trap 1 and 2 reflects higher sediment transport processes inside the new Inde River reach in comparison to the upstream part. Subdivision B (see Fig. 4) is morphodynamically active. The amount of sediment in sediment trap 3 is similar to that in sediment trap 2, 340 m^3^. The maximum amount of sediment, 860 m^3^, is trapped in sediment trap 6, which reflects significant morphodynamic developments in terms of the erosion of banks and the occurrence of sand banks and scour holes for subdivision F (see Fig. 4) during high-flow conditions [37]. Sediment trap 6 is no longer active. The same amount of sediment, 70 m^3^, is trapped in sediment traps 1 and 7 [37].

In sediment traps 2, 4, 6 and 7, the layering of the different materials and the development of an armor layer resulted in an increasing gravel fraction between 2005 AD and 2006 AD. In sediment traps 4, 6 and 7, the silt fraction increases significantly between 2005 AD and 2006 AD, and in sediment traps 1, 2, 3 and 5, the sand fraction increases significantly in 2006 AD in comparison to the reference sampling in 2005 AD [37].

An on-site inspection in 2008 AD shows that some sediment traps are completely filled up with sediment and, therefore, no longer active. Sediment is transported further downstream and is not captured inside the new Inde River reach, which results in an increased sediment input to the Rur River [42]. In 2008 AD and 2010 AD, sediment traps 6 and 7 were dredged to restore their functionality and to counteract the massive erosion of the new Inde River reach.

### Input and transport of heavy metals

**Fig. 13:**
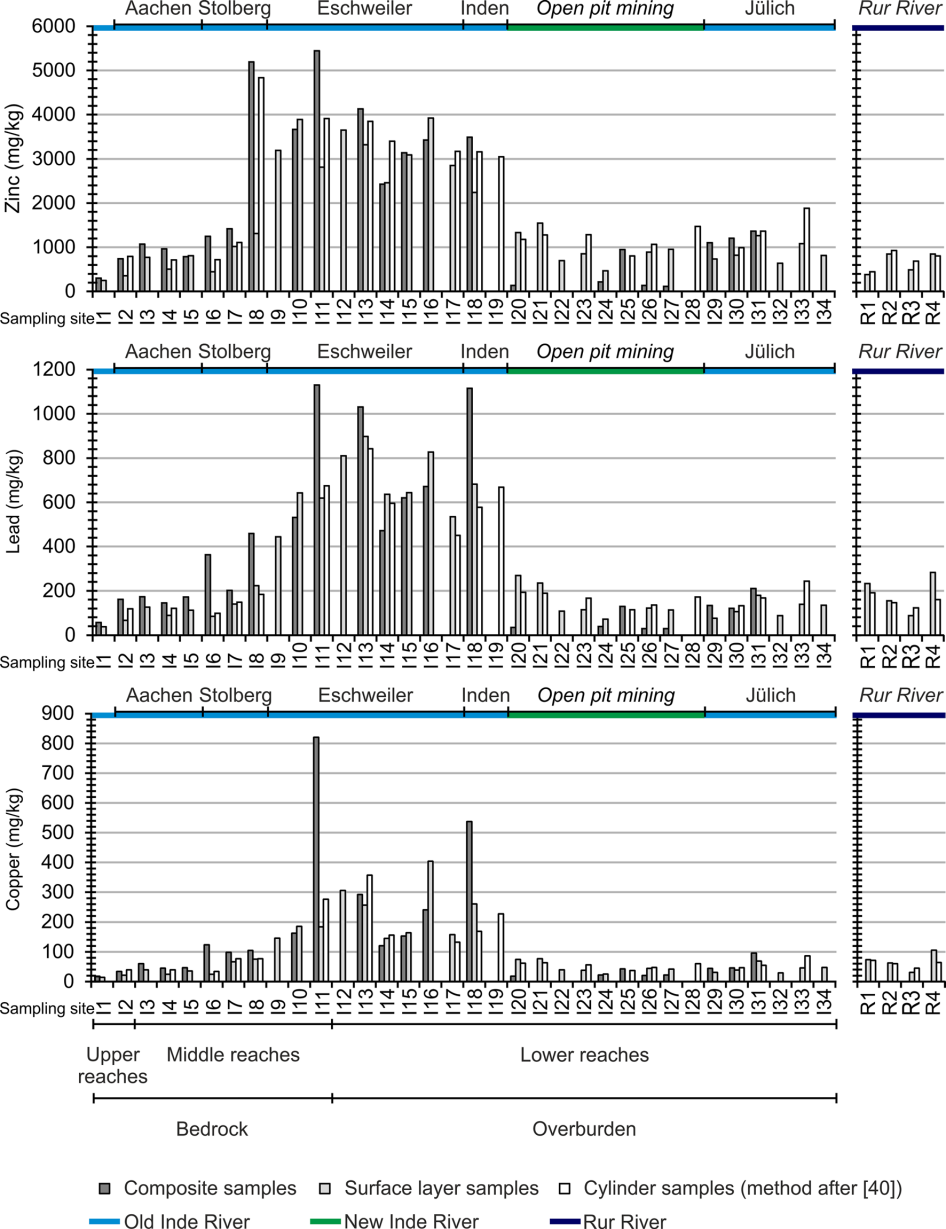
Zinc, lead and copper concentrations of Inde and Rur River (sites refer to Fig. 3)

Figure 13 shows the concentration profiles for zinc, lead and copper in the sediment samples used as a tracer for a further analysis of the morphological development. In the case of the missing bars, it was either not possible to sample the sediments, or to analyze them because of the grain size limitations of the X-ray fluorescence analysis.

The upper reaches and in part the middle reaches are, besides some past and recent anthropogenic impact factors, mainly characterized by the local geogenic background. There, in the samples of the surface layer, the concentrations of zinc range from 246 up to 1015 mg/kg, the content of lead from 38 to 140 mg/kg and of copper from 14 to 67 mg/kg (see Fig. 13, samples I1–I7).

The sediment samples of the surface layer I8–I18, collected in the middle reaches and in the lower reaches (see Fig. 13), show the highest content levels of the Inde River (Zinc: 3924 mg/kg, see Fig. 13, sample I16; Lead: 898 mg/kg, see Fig. 13, sample I13; Copper: 404 mg/kg, see Fig. 13, sample I16). Stolberg and Eschweiler were influenced by past ore mining and processing and coal mining as well as the iron and steel industry. Historical documents speculate about a beginning of mining in the study area in pre-Roman times. However, mining and ore processing are definitely postulated to have occurred since Roman times. In the sixteenth century, the master copper craftsmen left Aachen due to religious conflicts and moved to Stolberg. The number of brass manufacturing sites, hammer mills and wire manufacturers increased drastically [27, 43]. Coal mining between Stolberg and Eschweiler provided an energy resource, which was an important condition for the establishment of the iron and steel industry in the eithteenth century [44]. The closure of ore mining in 1919 AD was followed by the closure of the connected industries [33]. Currently, different factories still exist.

Even though the heavy metal concentrations of the surface layer samples collected from the new relocated riverbed (last part of the lower reaches, see Fig. 13, samples I20–I28) are significantly lower than those in the middle reaches and in parts of the lower reaches (see Fig. 13, samples I8–I19), they are higher compared to the concentrations analyzed in the samples collected in the upper reaches and partly in the middle reaches (see Fig. 13, samples I1–I7).

The surface layer sediments of the new riverbed show higher heavy metal contents than the composite samples of the upper 20 cm (see Fig. 13, samples I20–I28). An exception is the composite sample I25.

The XRF-analyses of four natural surface material samples (Forstkies) show average concentration of 73.19 mg/kg zinc, 21.99 mg/kg lead and 15.76 mg/kg copper. Due to the fact that the composite samples include morphologically active riverbed sediments with higher heavy metal enrichments in the upper part of the 20 cm, their concentration levels are slightly higher but comparable with the geogenic background of the heavy metal contents of the Forstkies samples. Thus, it is concluded that the lower part of the composite samples represents the natural background of the ground surface material (Forstkies), which was used for the construction of the new Inde River. In contrast to the other composite samples, I25 was taken in an accumulated sand bank. Its contents of zinc, lead and copper are comparable to the observations from the sediments that were suspended in the cylinder (method after [40]). Due to the recent formation of the sandbank, this sample does not represent the uncontaminated ground surface material (Forstkies), but an accumulation of surface layer sediments. The heavy metal contents of all the different sediment sample types show that the surface sediments in the whole new riverbed are characterized by enrichments of zinc, lead and copper (see Figs. 5, 13).

The results of the suspended sediments collected during bankfull discharge upstream of the restoration (same sampling site as I19, see Fig. 3) show concentrations of 2510.5 mg/kg zinc, 692.7 mg/kg lead and 124.4 mg/kg copper. These concentrations are higher than those observed in the sediment samples of the surface layer from the new riverbed. Surface layer sediment samples I20 and I21 show higher concentrations of heavy metals in contrast to samples I22–I27 (see Fig. 13). Samples I20 and I21 are closer to the old Inde River. The corresponding longitudinal profile of heavy metal concentrations between sampling sites I20 and I24 shows a slight decrease. The concentrations of samples I26 and I27 are higher and are comparable to those of sample I23 (see Fig. 13, samples I20–I27). Samples I26 and I27 belong to subdivision G (see Fig. 11). The available data show that this part is mainly characterized by the lowest erosion rates in the restoration during the first 3 years (275 m^3^/a or approx. 1 cm/a). Between 2008 AD and 2009 AD, the average sedimentation rate increased to a value of 3.500 m^3^/a (approx. 10 cm/a). Although a decrease in sedimentation took place between 2009 AD and 2010 AD (250 m^3^/a, approx. 1 cm/a) [41, 45], it is assumed that this subdivision was and still is characterized by sedimentation and thus by the accumulation of upstream eroded sediments and bounded heavy metals. The morphodynamic situation of sampling site I23 is similar.

Samples I29–I34 represent the reach of the old Inde River close to the mouth. Parts of this river section were changed due to the construction of another restoration (see Fig. 3).

The sediments that were suspended in the cylinder (method after [40]) represent the fraction that can potentially be remobilized from the riverbed during high discharges [40]. The sampled sediments show enrichments of heavy metals that are in general comparable with the surface layer samples (see Fig. 13, site I20, I21, I23, I25, I26 and I28).

Samples R1–R4 (see Figs. 3, 13) were sampled from the Rur River. R1 represents a sampling site upstream of the mouth of the Inde River. R2 is sampled directly inside the inflow of the Inde River into the Rur River. R3 and R4 are sampled 200 m (R3) and 300 m (R4) downstream of the mouth. The zinc content of R1 is lower than that of samples R2–R4. In the case of lead, sample R1 shows higher contents than the others, with the exception of the surface layer sediments of sampling site R4. Finally, the copper concentrations do not show clear differences. In all cases, sample R3 shows the lowest contents.

**Fig. 14:**
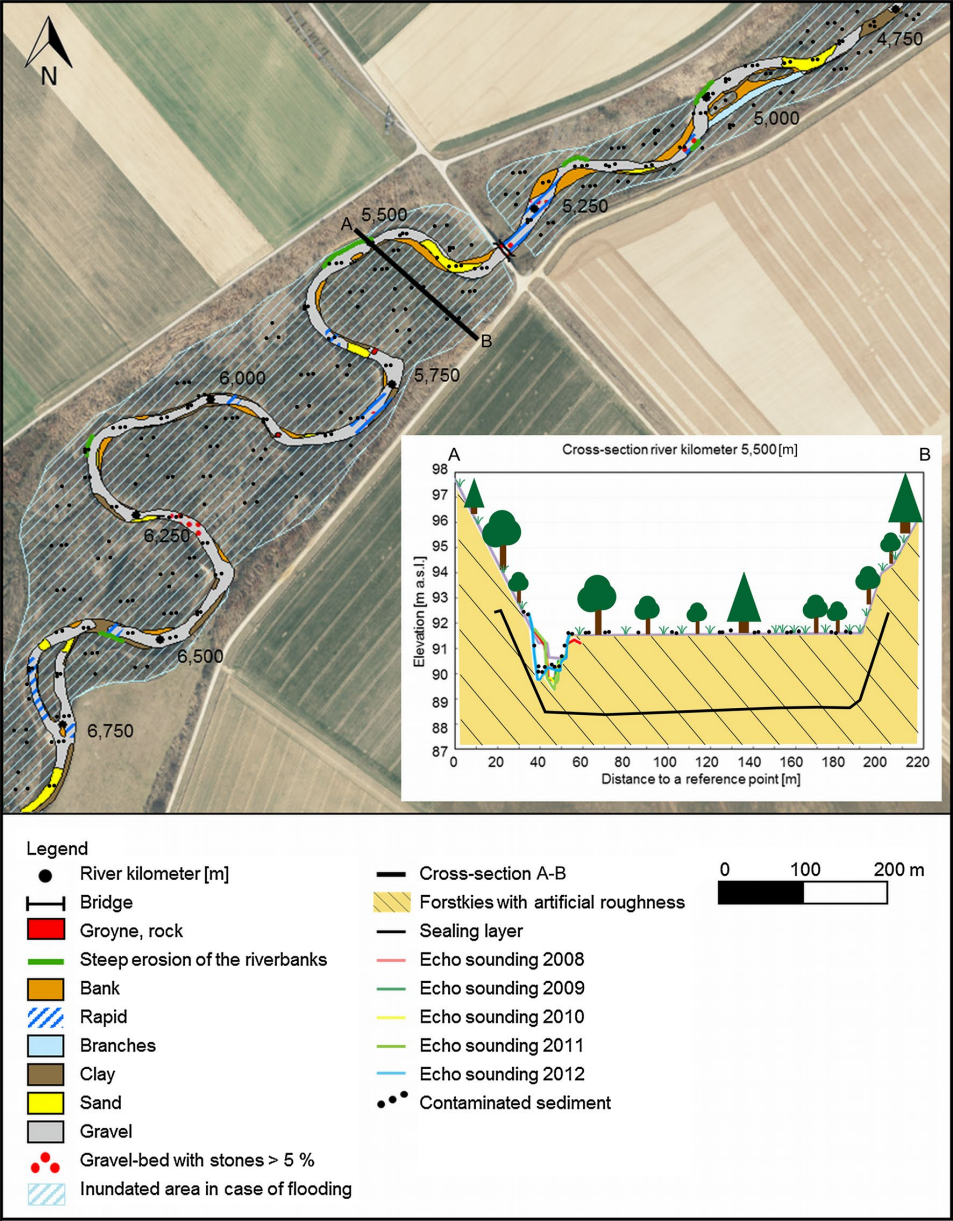
Fluvial morphodynamics of the new Inde River from the top view and an example cross-section

Figure 14 summarizes all the results and shows the fluvial morphodynamics of the new Inde River from the top view and an example cross-section.

## Discussion

At the Inde River, a river reach of 5 km was not only restored, but also relocated and enlarged to a 13-km-long reach and monitored over a time period of more than one decade. A restoration over such a length and a monitoring program over such a long time period focusing on the fluvial morphodynamics of the river are unique. Usually, restorations encompass only several hundred meters of a river. The restoration of the Emscher River, which started in 1990 AD, can be seen as an exception because it encompasses the entire course of the river. The Emscher River and its tributaries are converted from highly modified open wastewater channels with concrete beds to near-natural river systems in a large-scale restoration project of more than 30 years [46]. Such a conversion is not comparable to other restorations.

However, the monitoring of restorations deals mostly with comparisons of the pre- and immediate post restoration as case studies, but systematic studies to understand the evolution after the restoration over longer periods are missing [47]. The results of river restorations are predominantly evaluated focusing on the biotic response of the river (e.g. [48]). Nevertheless, the restoration of the Thur River in Switzerland [47] and the restoration of Willet Creek in Canada [49] also focus on the morphological development of the restored river reaches. At the Thur River, the morphodynamic evolution of the restored reaches of 2 km in length was investigated in relation to the role of pioneer vegetation roots in stabilizing the alluvial sediment [47]. At Willet Creek (restoration of 400 m flow length), a sediment budget for the watershed was developed, and the stability of the riffle-pool sequences established in the river and their effects on sediment transport processes were assessed [49]. Both studies were conducted over a time period of only 1 or 2 years, so that the results of the morphological development strongly depend on the discharges present during this time. A monitoring program of a time period of more than one decade could not be found in the literature, which underlines the uniqueness of the field measurements at the Inde River. Long-term monitoring programs should be performed to verify the restoration success and to filter out the local effects of natural variation [50].

The morphodynamic development of a relocated and restored river with the example of the new Inde River shows that sediment transport processes depend on different parameters and especially on the hydrological and geomorphological variability of the system as well as on the vegetation growth of the adjacent landscape. The vegetation is widespread and distinctive at the new Inde River and influences its morphological development intensively and over long time scales. In addition to the morphological development corridor [32] and the development towards the river’s guiding principle, the ecological passability and sediment continuity [51] of the new Inde River are two major benefits of the relocation and restoration. Here, achieving the guiding principles and ensuring the continuous transport of sediment through the river system mutually define each other.

The responsible water board of the Inde and Rur River (Wasserverband Eifel Rur) has measured an increasing fine sediment input of the Inde River into the Rur River after finishing the relocation and restoration in 2005 AD. A sudden and intensive transport of fine sediment in the downstream direction may cause the clogging of a downstream located riverbed, here the riverbed of the Rur River. Pores between gravel particles are clogged by clay, silt or fine sand [52], which reduces the spawning habitats of salmonids [53–55]. At the Inde River, there are different sources for sediment, which is eroded and then transported to the Rur River: (1) sediment eroded in the upper part of the Inde River, (2) sediment eroded from the riverbed inside the new Inde River reach, (3) sediment eroded from the floodplains inside the new Inde River reach and (4) sediment eroded from the adjacent hillsides inside the new Inde River reach (see Fig. 5).

### Concerning the sediment eroded upstream of the new Inde River reach

The riverbed is stabilized with approx. 44 bed drops, 4 bed slides, 41 bottom ramps and many bridges. Therefore, the continuous transport of sediment and sediment input from the upper part of the Inde River are limited. Until now, there have been no significant hydrological, morphological or geometrical changes in the upper part of the Inde River. The enrichment of the heavy metal content in the surface layer sediments indicate that due to fluvial morphodynamic processes, the new constructed riverbed of the new Inde River is characterized by ongoing and remarkable sediment and heavy metal inputs from upstream (see Fig. 5). The differences between the heavy metal contents of the sediment samples taken upstream and those within the restoration, as well as the analysis of the suspended sediments sampled during a bankfull discharge, verifiy that, in fact, the sediment input from upstream is characterized by higher heavy metal concentrations than those found in the analyzed riverbed sediments of the new Inde River.

### Concerning the sediment eroded from the riverbed and from the floodplains inside the new Inde River reach

Due to morphological processes in the riverbed, the sediments of the surface layer represent a mixture of uncontaminated natural ground surface material (Forstkies) and contaminated sediment input from upstream. Thus, due to dilution effects, the heavy metal contents in the new riverbed are lower than those upstream. Nevertheless, the surface sediments of the whole new riverbed show heavy metal enrichments. The distribution of sediment-bound heavy metals in the entire riverbed of the restoration relates to the sediment transport as well as its accumulation and indicates natural morphological processes and morphological development in the new Inde River. These results are also observed in the sedimentological characterization of the riverbed, in the analyses of the sediment traps and in the measured erosion and sedimentation rates of the riverbed (see Figs. 5, 10).

The substrate (Forstkies) used to prepare the riverbed of the new Inde River contains 72% sand and clay [56]. The grain size distribution of the riverbed is finer than the one postulated in the guiding principle of the Inde River and is eroded even during mean flow conditions, which results in an incision of the riverbed into the ground (see Fig. 5). In the first 5 years after finishing the restoration, a significant decrease in the fluvial morphodynamics of the Inde River occurred connected with a reduction of the incision and followed by an increase in the sedimentation in the whole restoration as shown in Figs. 11 and 12. This led towards an enrichment of heavy metals in the riverbed (see Fig. 5) and to a reduction of the dilution effect. Nevertheless, lower concentration levels of the composite samples compared with the sediments of the surface layer indicate, in general, rather low sedimentation processes or possibly even erosion during flood events.

The analysis of subdivision F (see Fig. 4) shows morphodynamic development in regard to the erosion of banks and the occurrence of sand banks and scour holes [37]. The heavy metal content of the composite sample I25 (see Fig. 13), which was taken in a sand bank, represents more recently accumulated sediments with heavy metal enrichments. The heavy metal contents are comparable with the sediments sampled with the cylinder method after [40] at this site. This proves that a morphodynamic analysis connected with heavy metals as a tracer is a valuable method to determine the development of the new Inde River. Future research will concentrate on the sedimentation and accumulation of contaminants on floodplains during overbank flood events.

### Concerning the sediment eroded from the adjacent hillsides inside the new Inde River reach

It was noticed during an on-site inspection in 2012 AD that there is a 5- to 10-cm-wide sediment deposition area at the bottom of the hillside, which is separated from the Inde River by an approx. 7-m-wide area with high vegetation. This indicates that the erosion of this hillside, which is also overgrown with vegetation, is decreased to almost zero and does not need any further analysis in the upcoming years. Flood events play an important role due to remobilization processes and higher erosion and accumulation rates [7, 8]. The analysis of a flood event in 2006 AD shows a deposition of 70 m^3^ of bed load in sediment trap 1 coming from upstream. Sediment traps 2–6 show even higher amounts of trapped sediments due to erosion and sediment transport inside the new Inde River. The sediments sampled with the cylinder method represent the fraction that can potentially be remobilized from the riverbed during flood events [40]. These remobilized sediments show enrichments of heavy metals. This supports the assumption that especially high discharges and flood events contribute to the heavy metal and sediment dispersion in the new riverbed. While the heavy metal concentrations in the first part of the restoration are especially influenced by a high sediment load from upstream, the remobilization, transport and sedimentation of sediment-bound heavy metals gain more importance with the increasing distance. This fact is also reflected in the data of predominating processes in the different subdivisions between 2011 AD and 2012 AD. Subdivision A (see Fig. 4), situated in the beginning of the restoration, is characterized by a sediment input from the upper part of the Inde River and by erosion, while subdivisions B to E (see Fig. 4) show, in general, accumulation processes.

The flood events of the years 2007 AD and 2008 AD resulted in an especially massive erosion of the riverbed [42] and an unnaturally high amount of fine sediment input to the Rur River. Although the heavy metal enrichment in the new riverbed is caused by a continuing sediment input from upstream, this sediment source and hillside erosion do not significantly increase the fine sediment input from the Inde River into the Rur River. Overall, this increased fine sediment input is definitively a consequence of the substrate used to prepare the riverbed of the new river reach (Forstkies).

Due to the heavy metal content of the Rur River sediments (see Fig. 13, sample R1) and dilution effects in the new Inde River, the Rur sediment samples (see Fig. 13, samples R2–R4) do not show significant increases in lead and copper downstream of the mouth of the Inde River. Only the differences of the zinc concentrations of samples R1–R4 let assume that the slight increase of zinc near the mouth is connected with the sediment input of the Inde River. The differences between the heavy metal concentrations of samples R2–R4 (see Fig. 13) can be explained by the morphodynamic processes and complex flow conditions connected with sediment transport and sedimentation in the Rur River at the mouth of the Inde River as well as with the different grain size compositions of the samples.

However, the results for the years 2010 AD until 2012 AD show that the massive erosion inside the new Inde River reach decreased towards almost zero (see Fig. 11). It is expected that the erosion will decrease with the increasing age of the new Inde River. Because of the decreasing erosion in the riverbed and on the adjacent hillsides, the accumulation of sediment-bound heavy metals increases in the restoration. This process will continue due to ongoing natural morphodynamic processes.

## Conclusion

The fluvial morphodynamics of a new river reach of the Inde River (North-Rhine Westphalia, Germany) were determined after its relocation and restoration in 2005 AD with extensive field measurements between 2005 AD and 2018 AD. The results of the analysis showed the incision of the new riverbed between 2005 AD and 2008 AD as well as a downstream movement of sediments, resulted in a massive sediment input at the mouth of the Inde River into the Rur River. With these morphological processes, which are connected with flood events and vegetation growth, the river system adapted its new conditions. Morphological processes decelerated with the increasing vegetation growth. The flow velocities, grain size distributions of the riverbed and occurrence of fluvial forms such as banks and branches are very variable.

In 2018 AD, the new Inde River has reached a dynamic morphological equilibrium, which has improved and still improves its natural hydrological and morphological conditions as an ecological habitat. Between 2008 AD and 2013 AD, the riverbed incision significantly decreased. Therefore, after almost 13 years of adaptation processes, the Inde River itself represents a dynamic equilibrium, and its new surrounding landscape has developed towards a quasi-stable system.

Currently, the predominating sedimentation processes in the river system are accompanied by an accumulation of sediment-bound heavy metals. Overall, 13 years after connecting the relocation and restoration to the consisting river reaches, the sediment samples collected from the new riverbed show considerable heavy metal concentrations (see Fig. 14).

It is concluded that the new Inde River has developed towards its pre-defined guiding principle within its anthropogenic restrictions caused by the open-pit lignite mine Inden and that it has reached its goal of being a natural river. Especially due to the length (13 km) of the new constructed riverbed, the Inde River is a very good study area and a representative example showing that achieving a dynamic morphological equilibrium after a river relocation and restoration is possible after only one decade.

The results can generally be used for further investigations on fluvial morphodynamics. Overall, the results show the following:

- A new constructed riverbed of a river restoration or relocation can be used to analyze the downstream movement of sediments.
- Contaminated sediments can be used as a tracer for morphological development.
- The morphological adaptation processes of a new restoration are due to hydrological and vegetation development and that
- A dynamic equilibrium can be reached only after a few years of adaptation processes, which were characterized by remarkable erosion and accumulation.
